# Differing Roles for *TCF4* and *COL8A2* in Central Corneal Thickness and Fuchs Endothelial Corneal Dystrophy

**DOI:** 10.1371/journal.pone.0046742

**Published:** 2012-10-23

**Authors:** Robert P. Igo, Laura J. Kopplin, Peronne Joseph, Barbara Truitt, Jeremy Fondran, David Bardenstein, Anthony J. Aldave, Christopher R. Croasdale, Marianne O. Price, Miriam Rosenwasser, Jonathan H. Lass, Sudha K. Iyengar, for the FECD Genetics Multi-center Study Group

**Affiliations:** 1 Department of Epidemiology and Biostatistics, Case Western Reserve University, Cleveland, Ohio, United States of America; 2 Department of Ophthalmology, Casey Eye Institute, Portland, Oregon, United States of America; 3 Department of Ophthalmology and Visual Sciences, Case Western Reserve University and University Hospitals Eye Institute, Cleveland, Ohio, United States of America; 4 The Jules Stein Eye Institute and the Department of Ophthalmology, David Geffen School of Medicine at University of California, Los Angeles, Los Angeles, California, United States of America; 5 David Duehr Dean Clinic, Madison, Wisconsin, United States of America; 6 Price Vision Group, Indianapolis, Indiana, United States of America; 7 Central Pennsylvania Eye Institute, Hershey, Pennsylvania, United States of America; 8 Department of Genetics, Case Western Reserve University, Cleveland, Ohio, United States of America; Radboud University Nijmegen Medical Centre, Netherlands

## Abstract

Fuchs endothelial corneal dystrophy (FECD) is the most common late-onset, vision-threatening corneal dystrophy in the United States, affecting about 4% of the population. Advanced FECD involves a thickening of the cornea from stromal edema and changes in Descemet membrane. To understand the relationship between FECD and central corneal thickness (CCT), we characterized common genetic variation in *COL8A2* and *TCF4*, genes previously implicated in CCT and/or FECD. Other genes previously associated with FECD (*PITX2, ZEB1, SLC4A11)*, and genes only known to affect CCT (*COL5A1, FOXO1, AVGR8, ZNF469*) were also interrogated. FECD probands, relatives and controls were recruited from 32 clinical sites; a total of 532 cases and 204 controls were genotyped and tested for association of FECD case/control status, a 7-step FECD severity scale and CCT, adjusting for age and sex. Association of FECD grade with *TCF4* was highly significant (OR  = 6.01 at rs613872; *p* = 4.8×10^−25^), and remained significant when adjusted for changes in CCT (OR  = 4.84; *p* = 2.2×10^−16^). Association of CCT with *TCF4* was also significant (*p* = 6.1×10^−7^), but was abolished with adjustment for FECD grade (*p* = 0.92). After adjusting for FECD grade, markers in other genes examined were modestly associated (*p* ∼ 0.001) with FECD and/or CCT. Thus, common variants in *TCF4* appear to influence FECD directly, and CCT secondarily via FECD. Additionally, changes in corneal thickness due to the effect of other loci may modify disease severity, age-at-onset, or other biomechanical characteristics.

## Introduction

The curvature, thickness, and function of the cornea are controlled by the fine structure of its five layers. Fuchs endothelial corneal dystrophy (FECD), which results in loss of vision associated with progressive corneal edema and loss of corneal transparency, is estimated to have a prevalence of approximately 4% in the United States [1], [2], where it is one of the most common indications for corneal transplantation [3]. The principal defect in FECD is a decline in the number of functional corneal endothelial cells, with compensatory abnormalities, such as thickening of Descemet membrane, and subsequent thickening of the cornea due to edema. Thus, individuals with clinically advanced FECD have measurably thicker central corneas. In the initial stages of the disease, excrescences form on Descemet membrane along with deposition of abnormal, excess collagen posterior to the membrane (the “posterior collagenous layer”), resulting in the clinical and pathologic appearance of guttae [4]. Compromise of endothelial function may result in corneal stromal edema, epithelial edema, and painful bullous keratopathy. Penetrating or endothelial keratoplasty is the only definitive treatment, with palliative care the only option prior to surgery.

The pathophysiology underlying the classic, late-onset form of FECD remains unknown [5], [6], although genetic predisposition has been reported as the most reliable risk factor for disease [1], [7]–[12]. As in many other common complex diseases, it is widely recognized that genetic susceptibility to FECD is determined by variants in many genes (Table 1). It is not known whether these genes act in concert or independently. Variants in two genes, type VIII collagen, alpha 2 subunit (*COL8A2*) [13], [14], and transcription factor 4 (*TCF4*) [15]–[19], have consistently shown association with FECD. Mutations in *COL8A2* are rare in patient populations [20], [21], but some, e.g., the missense mutations Leu450Trp and Gln455Lys, cause highly penetrant, early-onset, forms of the disease associated with thickening of Descemet membrane and subsequent increase in central corneal thickness (CCT) [13], [14], [22]. Other rare genetic variants in *COL8A2*, however, are associated with corneal thinning in a Caucasian sample [23], as were common variants in *COL8A2* in an Asian sample [24]; and corneal thinning was also associated with loss of *COL8A2* in animal models [25], [26]. By contrast, non-coding common variants, e.g., rs613872, in *TCF4* show strong association with disease, but it is unclear whether these variants directly mediate pathology [15]–[19].

**Table 1:** Candidate genes for FECD and related diseases of the cornea.

Location	Gene	FECD	CCT	Associated conditions
1p34.1	*COL8A2*	Early-onset [13], [14], [39], [40]	Decreased [23], [24]	PPCD [13]; POAG [23]
4q25	*PITX2*	Early-onset (with A-R syndrome) [30]	Decreased [31]	A-R syndrome [30], [31]
9q34.3	*COL5A1*		Decreased [24], [33]	
10p11.22	*ZEB1*	Late-onset [36], [56]		PPCD [35], [41]–[43]
13q12.11	*AVGR8*		Decreased [33]	
13q14.11	*FOXO1*		Increased [32], [33]	
14q12	*AKAP6*		Increased [24]	
15q25.3	*AKAP13*		Increased [33]	
16q24.2	*ZNF469*		Decreased [24], [32], [33]	BCS [46], [47]
18q21.2	*TCF4*	Late-onset [15]–[19]		
20p13	*SLC4A11*	Late-onset [27], [38]		CHED2 [37], [44], [45], CDPD [67]

The columns FECD and CCT indicate whether genes have been implicated in Fuchs dystrophy and central corneal thickness, respectively. The effects on CCT are those for rare (variant) alleles; “Increased” and “Decreased” indicate that the variant (minor) allele is associated with an increase or decrease in CCT. A-R syndrome, Axenfeld-Rieger syndrome; PPCD, posterior polymorphous corneal dystrophy; POAG, primary open angle glaucoma; CHED, congenital hereditary endothelial dystrophy; CDPD, corneal dystrophy and perceptive deafness (Harboyan syndrome).

These results point to shared genetic determinants for FECD, CCT and potentially other corneal endothelial dystrophies, acting to disorganize the corneal architecture. Some investigators have postulated that there is a continuum of disorders between many of the corneal endothelial dystrophies including posterior polymorphous corneal dystrophy (PPCD), congenital hereditary endothelial dystrophy (CHED) and FECD [27], all of which share features of thickening the cornea [28], . Given that we examined genes for all three disorders, we also examined the phenotype of CCT to determine if these genes do play a role in Fuchs dystrophy by moderating the thickness or thinness of the cornea. Mutations in *PITX2*, a developmental regulatory gene, have shown association with FECD [30], corneal thinning [31] and Axenfeld-Rieger syndrome [30], [31]. Several other genes have been identified through genetic association studies as potential determinants of CCT [24], [32], [33], but have not been extensively tested in FECD patients (Table 1).

Here, we report results from an association analysis on a large sample of late-onset FECD cases and controls from the FECD Genetics Multi-center Study Group [10]. We surveyed common genetic variation in nine genes that may contribute to CCT, FECD and related corneal dystrophies for association with three phenotypes: FECD case-control status, a seven-step semi-quantitative FECD severity scale, and CCT. To distinguish direct effects on CCT from corneal thickening secondary to FECD, we adjusted for CCT in analyses of FECD, and vice versa.

## Materials and Methods

### Ethics Statement

This work was performed in accordance with the tenets of the Declaration of Helsinki. Written informed consent was obtained from all participants. Data were collected under multi-center Institutional Review Board (IRB) approval.

### Study sample

Families enriched in FECD and unrelated FECD controls were recruited by the FECD Genetics Multi-center Study, as previously described [10]. Only individuals of European descent were genotyped for this study, to minimize population heterogeneity; ethnic classification was self-reported.

The severity of FECD was assessed using a modified grading scale [1] with grades from 0 to 6. FECD controls were required to have no evidence of FECD (grade 0 in both eyes, indicating lack of guttae), whereas cases were defined as having a grade of 4 (2–5 mm confluent guttae) or higher in the worse eye. Participants with grades 1–3 in the worse eye were considered missing for FECD case/control status, but were included in analyses of FECD grade (as intermediate cases) and CCT. Investigators were provided a grading scale with photographic examples of each grade, a training manual and video demonstration of slit-lamp biomicroscopy. A certification exam for grading FECD was also administered to investigators at the outset of the study (for details, see [10]).

Probands eligible for recruitment had an available histopathology specimen, containing the endothelial layer and Descemet membrane, from penetrating or endothelial keratoplasty; a pathology report; or a family member with advanced FECD confirmed by histopathological examination. Families containing a proband and a living relative (other than parents or children) were eligible for inclusion. Control subjects were matched to index cases by age, sex and ethnic group, and were required to have an FECD grade of 0 in both eyes.

Eyes of FECD cases and relatives were excluded from the study for any of the following conditions: cataract surgery within one year of examination; history of blunt, penetrating or perforating trauma to the cornea; or evidence of another corneal endothelial dystrophy. In addition, eyes that had undergone penetrating or endothelial keratoplasty were excluded from analyses involving CCT. Exclusion criteria for unrelated controls included family history of heritable disease of the cornea, bilateral corneal surgery, perforating corneal trauma with scarring, refractive astigmatism of greater than 3.5 diopters, or a history of interstitial or infectious keratitis, anterior uveitis, or vascularization of the cornea.

CCT measurements were obtained by ultrasonic pachymetry. Pachymeters were used from the following manufacturers: Accutome, Malvern PA; Bausch and Lomb Surgical, Rancho Cucamonga, CA; DGH Technology, Exton PA; KMI Surgical, Downington, PA; Eye Technology, Inc., Ardmore, PA; Haag-Streit, Mason, OH; Sonogage, Cleveland, OH; Sonomed, New Berlin, WI; and Tomey, Phoenix, AZ.

### Genotyping

SNPs were chosen from published reports on the genetics of FECD and related diseases of the cornea [14], [15], [30], [32], [34]–[38]. We selected two genes that have previously been associated with FECD: *COL8A2* [13], [14], [39], [40] and *TCF4* [15]–[19]. We also selected genes predominantly associated with PPCD (*ZEB1*) [35], [41]–[43], CHED (*SLC4A11*) [37], [44], [45], Axenfeld-Reiger Syndrome (*PITX2*) [30], [31], brittle cornea syndrome (*ZNF469*) [46], [47] and with central corneal thickness (*COL5A1* [24], [33], *AVGR8* [33], *FOXO1* [32], [33], *AKAP6* [24], and *AKAP13* [33]).

For the genes *COL8A2, ZEB1* (*TCF8*), *PITX2* and *SLC4A11*, additional SNPs were selected to capture common genetic variation, using Tagger [48]. Tag SNPs were identified under an *r* ^2^ threshold of 0.8, based on the linkage disequilibrium in the HapMap CEU sample. In addition, some SNPs were chosen from regions highly conserved among mammals.

DNA samples were obtained from study participants as described [10]. Samples were genotyped for candidate SNPs via two assays: TaqMan (Applied Biosystems, Carlsbad, CA) and LGC Genomics (formerly KBiosciences, Teddington, Middlesex, UK). For each assay, genotypes were automatically called by the Applied Biosystems Sequence Detection System software, and afterwards, clusters were manually reviewed. Twelve TaqMan assays with rare minor alleles were clustered manually. One TaqMan SNP was found to harbor a DNA copy-number variant on manual review, and was not used for SNP association analysis. For LGC Genomics genotyping, SNPs were automatically called via the KASP system (http://www.kbioscience.co.uk/reagents/KASP.html) and viewed with LGC's SNP Viewer software. The KASP assay uses fluorescent resonance energy transfer to suppress (quench) fluorescence in reporter oligonucleotides until they are incorporated into allele-specific PCR products.

Approximately 0.26% of the genotypes were manually called after inspection of the clusters. For both genotyping methods, SNPs were designed in-house and were validated by the respective companies. Ten DNA samples were omitted for analysis due to poor genotyping (call rate <95%). Eighteen SNPs with rare minor alleles (frequency <1% in the FECD sample) were not analyzed for SNP association.

Genotype data on 1092 individuals were downloaded from the 1000 Genomes Project database (http://www.1000genomes.org/; accessed February 1, 2012), October 2011 Integrated Variant Set (release ICHG2011), using the Data Slicer tool. Six individuals were omitted from calculations of allele frequencies because of family relationships with other members of the data set.

### Statistical Analysis

Allele frequencies in FECD cases and controls were estimated by the maximum-likelihood approach for family data implemented in the FREQ program in the S.A.G.E. software package [49].

Association analysis with adjustment for family correlations was carried out using the GWAF package for R [50]. GWAF fits a linear mixed model for quantitative traits in which the SNP genotype is included as a fixed effect. Family correlations are modeled as a polygenic random effect. For binary (case/control) traits, GWAF estimates the correlation structure among relatives via a generalized-estimating-equation (GEE) model within the logistic regression framework. Age and sex were included as covariates in all analyses. In some analyses, the semi-quantitative FECD severity grade was included as a covariate affecting CCT, and vice versa. Association analyses incorporated an additive genetic model, with each minor allele exerting the same effect, unless fewer than ten individuals with the rare homozygous genotype were available, in which case the dominant model in the rare allele was used. Gene-by-gene interactions were coded as the product of the number of minor alleles at each SNP in the model. Meta-analysis combining results from published studies was conducted by inverse-variance-weighted averaging of log ORs. Adjustment for multiple testing was carried out through a Bonferroni correction based on the number of effective independent tests accounting for LD structure among SNPs in the same gene, as determined by the program SNPSpDlite (http://gump.qimr.edu.au/general/daleN/SNPSpDlite/) [51] using the estimate proposed by Li and Ji [52].

In haplotype-based association analyses, a subset of the genotyped sample comprising unrelated cases (mostly probands) and controls was selected (*n* = 245 cases and 158 controls). Haplotype frequencies and effects were estimated by linear regression with concurrent haplotype phasing, using the haplo.glm function of the R package HaploStats [53], and overall significance was measured by a score test implemented under the haplo.score function.

## Results

### Study sample

A total of 531 FECD cases with clinically significant disease and 204 controls were successfully genotyped (Table 2); 87 individuals had intermediate FECD status. Although a substantially greater proportion of cases were female (71%, vs. 54% in controls), the cases and controls were well matched in age (*p*>0.05 for difference in mean age, by Student's *t* test). CCT in cases was overall significantly greater than that in controls (*p*<10^−6^), with a mean thickness 55 µm greater.

**Table 2:** Summary of genotyped samples.

	All	FECD Cases	FECD Controls
*n* a	822	531	204
Female, *n* (%)	545 (66.3%)	377 (71.0%)	154 (54.4%)
Age	66±12	67±12	67±10
FECD Grade	3.5±2.3	5.1±0.7	0.0±0.0
CCT^b^	589±59	611±62	556±39

Unless otherwise indicated, statistics are shown as mean ± SD.

a Total includes 87 individuals with FECD grade of 1–3 in worse eye, not classified as FECD cases or controls.

b Average of two eyes, when available.

### Association results for FECD case/control status and FECD grade

We found a highly significant association between FECD case/control status and SNP rs613872 in *TCF4* (*p* = 2.0×10^−19^; Table 3), confirming results from earlier studies [15]–[19]. The per-allele odds ratio (OR) of 6.01 is consistent with the large ORs reported originally in a Caucasian sample [15]. Consistent with this large effect, the frequency of the risk, or G, allele was much greater in cases (0.477) than in controls (0.191). The association remained very strong when FECD status was adjusted for CCT (*p* = 2.0×10^−13^), with an allelic OR of 4.84. We observed two other nominally significant associations with the binary FECD trait, one SNP each in pituitary homeobox 2 (*PITX2*) (rs17554590) and autogenous vein graft remodeling associated protein 8 (*AVGR8*) (rs1034200), with *p* values of 0.028 and 0.015, respectively, for FECD status adjusted for CCT (Table 3 and Table S1).

**Table 3:** Association analyses for FECD case/control status.

						FECD only		Adjusted for CCT	
Gene	Chr	SNPs	Best SNP	Position	Ref. All.	OR (95% CI)	p	OR (95% CI)	p
*COL8A2*	1	15	rs7553155	36,568,236	A	0.72 (0.46, 1.13)*	0.19*	0.86 (0.50, 1.46)*	0.57*
*PITX2*	4	18	rs17554590	111,562,902	G	2.12 (0.91, 4.91)	0.081	2.70 (1.11, 6.57)	*0.028*
*COL5A1*	9	4	rs1536478	137,432,248	T	1.15 (0.88, 1.48)	0.30	1.07 (0.77, 1.47)	0.69
*ZEB1*	10	25	rs11008516	31,819,360	G	1.27 (0.88, 1.84)	0.20	1.23 (0.80, 1.88)	0.34
*AVGR8*	13	1	rs1034200	23,228,691	T	0.82 (0.62, 1.07)	0.14	0.66 (0.47, 0.92)	*0.015*
*FOXO1*	13	1	rs2755237	41,109,429	C	1.07 (0.75, 1.52)	0.72	1.38 (0.90, 2.12)	0.14
*AKAP6*	14	1	rs768787	33,046,471	T	0.99 (0.76, 1.27)	0.92	0.99 (0.71, 1.37)	0.96
*AKAP13*	15	1	rs6496932	85,825,567	A	0.82 (0.60, 1.12)	0.21	0.92 (0.60, 1.42)	0.71
*ZNF469*	16	5	rs9925231	88,338,107	T	0.96 (0.74, 1.23)	0.72	1.09 (0.81, 1.48)	0.56
*TCF4*	18	1	rs613872	53,210,302	G	6.01 (4.07, 8.87)	**2.0×10^−19^**	4.84 (3.18, 7.37)	**2.0×10^−13^**
*SLC4A11*	20	17	rs2144771	3,214,020	G	0.88 (0.68, 1.13)	0.32	0.82 (0.61, 1.11)	0.20

Chr., chromosome; No. SNPs, number of SNPs in or near gene passing QC; Best SNP, SNP with smallest *p* value; Position, physical map position (NCBI human genome build 36); Ref. All., reference (minor) allele; OR, odds ratio per copy of the reference allele (additive model) or for presence of minor allele (dominant model). *p* values in *italics* are less than 0.05; in **bold,** less than 0.001. *, dominant model.

We obtained similar results from association analyses on the seven-step FECD severity scale taken as a quantitative trait (Table 4). The rs613872 variant in *TCF4* was highly significantly associated with FECD severity grade, whether adjusted for age and sex only (*p* = 4.8×10^−25^) or whether CCT was included as a covariate (*p* = 2.2×10^−16^). Each G allele of rs613872 was associated with a 1.15-unit increase in FECD severity grade when adjusted for age and sex only, and with a 1.02-unit increase when adjusted further for CCT in the eye with more severe FECD. Variation in rs613872 accounted for 10.9% and 9.6% of the variance in CCT and CCT adjusted for FECD, respectively. Results for other variants were, in general, not significant (Table S2). However, the *PITX2* SNP rs17554590 and *AVGR8* SNP rs1034200 showed modest associations with FECD severity (*p* = 0.0073 and 0.019, respectively), as did one marker in zinc-finger protein 469 (*ZNF469*) (*p* = 0.0081), after adjustment for CCT (Table 4). These markers explained 0.6% (*PITX2*), 0.6% (*AVGR8*) and 0.4% (*ZNF469*) of the phenotype variance, respectively (Table S2). No SNP apart from rs613872 in *TCF4* reach a level of significance of *p*<0.0010, the Bonferroni-corrected threshold for an experiment-wide significance of 0.05 based on an effective number of 48 independent SNPs across all the genes, as determined by analysis of correlation among SNPs [51] (see Materials and Methods).

**Table 4:** Association analyses for FECD severity grade (worse eye).

						Grade only		Adjusted for CCT	
Gene	Chr.	SNPs	Best SNP	Position	Ref. All.	Effect (95% CI)	P	Effect (95% CI)	p
*COL8A2*	1	15	rs7542594	36,593,856	A	−0.23 (−0.47, 0.01)	0.063*	0.09 (−0.17, 0.36)*	0.50*
*PITX2*	4	18	rs17554590	111,562,902	G	0.18 (−0.46, 0.82)	0.57*	0.96 (0.26, 1.66)	0.0073*
*COL5A1*	9	4	rs4840244	137,431,904	T	0.07 (−0.14, 0.29)	0.51	0.00 (−0.24, 0.24)	0.97
*ZEB1*	10	25	rs12217563	31,741,273	C	0.27 (0.04, 0.49)	*0.020*	−0.02 (−0.28, 0.24)	0.87
*AVGR8*	13	1	rs1034200	23,228,691	T	−0.04 (−0.27, 0.19)	0.72	−0.35 (−0.60, −0.09)	*0.0082*
*FOXO1*	13	1	rs2755237	41,109,429	C	0.07 (−0.21, 0.35)	0.62	0.22 (−0.10, 0.53)	0.18
*AKAP6*	14	1	rs768787	33,046,471	T	−0.12 (−0.32, 0.08)	0.25	−0.04 (−0.26, 0.18)	0.73
*AKAP13*	15	1	rs6496932	85,825,567	A	−0.20 (−0.45, 0.06)	0.14	−0.12 (−0.41, 0.17)	0.41
*ZNF469*	16	5	rs9927272	88,346,709	G	0.12 (−0.08, 0.33)	0.24	0.25 (0.03, 0.47)	*0.024*
*TCF4*	18	1	rs613872	53,210,302	G	1.15 (0.93, 1.36)	**4.8×10** ^−**25**^	1.02 (0.78, 1.27)	**2.2×10** ^−**16**^
*SLC4A11*	20	17	rs2422862	3,199,703	G	0.23 (−0.04, 0.51)	0.097	0.11 (−0.20, 0.43)	0.48

Chr., chromosome; No. SNPs, number of SNPs in or near gene passing QC; Best SNP, SNP with smallest *p* value; Position, physical map position (NCBI human genome build 36); Ref. All., reference (minor) allele; effect, expected change in FECD grade per copy of the reference allele (additive model) or for presence of minor allele (dominant model). *p* values in *italics* are less than 0.05; in **bold,** less than 0.001 (the Bonferroni threshold for study-wide significance at the 0.05 level). *, dominant model.

### Meta-analysis for association of *TCF4* SNP rs613872 with FECD

**Figure 1:**
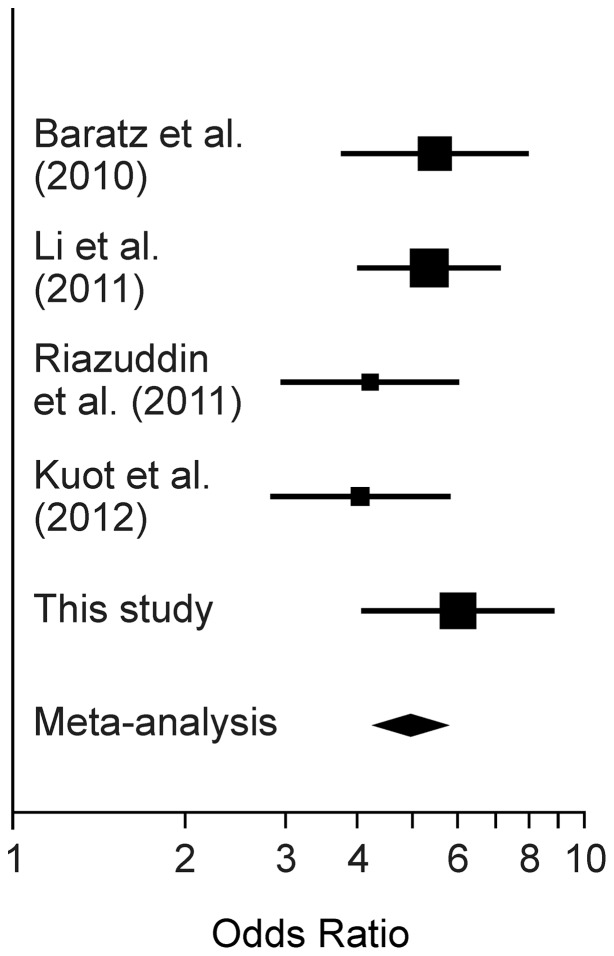
Meta-analysis for association of *TCF4* SNP rs613872 with FECD case/control status. Horizontal lines denote the 95% CI for the OR for each G allele at rs613872. The width of the squares indicating estimates of OR is proportional to the sample size in each study.

We combined our association result for rs613872 with data from previously published studies on Caucasian populations [15]–[17], [19] in an inverse-variance-weighted meta-analysis (Fig. 1). Sample sizes in the previous studies ranged from 350 to 790 combined FECD cases and controls. The combined OR for each G allele was 4.96 (95% CI  =  [4.25, 5.80]; *p* = 2.0×10^−89^).

### Association results for CCT

In contrast to the FECD severity grade, association analyses for CCT yielded significant results on a large number of SNPs in several genes (Table 5 and Table S3). The G allele of rs613872 in *TCF4* was very strongly associated with increasing corneal thickness (*p* = 6.1×10^−7^; Bonferroni-corrected *p* = 2.9×10^−5^), with each copy conferring an expected 18.6-µm increase in CCT. Eleven SNPs in or near *COL8A2* show weak evidence for association with *p*<0.05, with one study-wide significant result with regard to rs4652900 (*p* = 7.8×10^−4^; Bonferroni-corrected *p* = 0.037). Each copy of the minor allele of rs4652900 was associated with a 14.8-µm decrease in CCT (95% CI  =  [−23.4, −6.2]). An analysis of interaction between *TCF4* SNP rs613872 and each of the SNPs in *COL8A2*, including additive main effects and an interaction term, failed to detect significant gene-by-gene interaction effects (*p*>0.10 in all tests).

**Table 5:** Nominally significant results from association analyses for CCT.

Gene/SNP	Position	Ref.	Effect (95% CI)	p	Effect (95% CI)	p
*COL8A2*						
rs491603	36,532,316	T	−13.1 (−22.1, −4.1)	*0.0043*	−10.4 (−18.2, −2.6)	*0.0090*
rs538638	36,534,644	G	−13.4 (−26.5, −0.4)*	0.043*	−11.0 (−22.2, 0.2)*	0.055*
rs3754076	36,549,157	A	−14.0 (−27.1, −0.9)*	0.036*	−13.1 (−24.4, −1.9)*	0.022*
rs3767703	36,555,758	T	−17.6 (−31.5, −3.6)*	0.014*	−13.1 (−25.2, −1.0)*	0.033*
rs3738360	36,563,158	C	−15.1 (−28.3, −1.8)*	0.026*	−14.6 (−26.0, −3.2)*	0.012*
rs274754	36,565,617	G	−11.0 (−19.3, −2.7)	*0.0095*	−8.0 (−15.1, −0.8)	*0.030*
rs4652900	36,565,999	A	−14.8 (−23.4, −6.2)	**0.00078**	−11.6 (−19.1, −4.2)	*0.0023*
rs7550047	36,567,343	G	−16.8 (−29.9, −3.7)*	0.012*	−11.8 (−23.1, −0.5)*	0.049*
rs7553155	36,568,236	A	−16.3 (−28.7, −3.9)*	0.010*	−10.8 (−21.5, −0.0)*	0.049*
rs96067	36,571,920	G	−14.0 (−23.0, −5.1)	*0.0020*	−10.0 (−17.7, −2.4)	*0.010*
rs7542594	36,593,856	A	−11.3 (−19.4, −3.2)	*0.0062*	−9.0 (−16.0, −2.0)	*0.012*
*PITX2*						
rs1947187	111,545,497	T	−9.5 (−18.4, −0.6)	*0.037*	−7.7 (−15.4, −0.0)	*0.049*
*COL5A1*						
rs1409832	137,428,425	G	−8.7 (−16.4, −1.0)	*0.027*	−7.8 (−14.4, −1.2)	*0.021*
*ZEB1*						
rs12217563	31,741,273	C	7.9 (0.3, 15.5)	*0.042*	6.8 (0.1, 13.4)	*0.047*
rs12781751	31,912,832	C	8.4 (1.7, 15.1)	*0.014*	7.0 (1.2, 12.8)	*0.018*
rs10763867	31,914,975	A	7.2 (0.5, 13.8)	*0.035*	7.3 (1.5, 13.0)	*0.013*
rs4747753	31,917,426	T	8.4 (1.5, 15.2)	*0.016*	7.3 (1.4, 13.2)	*0.015*
rs7092560	31,925,230	G	10.0 (2.6, 17.3)	*0.0079*	9.8 (3.4, 16.1)	*0.0025*
*AVGR8*						
rs1034200	23,228,691	T	6.7 (−0.9, 14.4)	*0.084*	9.8 (3.2, 16.4)	*0.0035*
*ZNF469*						
rs12447690	88,298,124	C	−6.6 (−13.5, 0.3)	0.059	−6.9 (−12.8, −0.9)	*0.039*
rs9938149	88,331,640	C	−7.7 (−14.3, −1.0)	*0.024*	−7.1 (−12.9, −1.4)	*0.015*
rs9927272	88,346,709	G	−4.3 (−10.9, 2.4)	0.21	−5.8 (−11.6, −0.1)	*0.047*
*TCF4*						
rs613872	53,210,302	G	18.6 (11.3, 25.9)	**6.1×10^−7^**	−0.4 (−7.4, 6.6)	0.92
*SLC4A11*						
rs6084312	3,211,235	T	14.2 (2.7, 25.8)*	0.016*	13.7 (3.8, 23.5)	0.0068*
rs3803955	3,214,126	T	−10.5 (−18.1, −3.0)	*0.0064*	−10.8 (−17.4, −4.3)	*0.0012*
rs6051669	3,216,203	T	−8.6 (−15.2, −2.0)	*0.011*	−8.5 (−14.3, −2.8)	*0.0036*

Genes *FOXO1, AKAP6* and *AKAP13* showed no significant associations, and therefore do not appear in this table. Position, physical map position (NCBI human genome build 36); Ref. All., reference (minor) allele; effect, expected µm change in CCT per copy of the reference allele (additive model) or for presence of minor allele (dominant model). *p* values in *italics* are less than 0.05; in **bold,** less than 0.001 (the Bonferroni threshold for study-wide significance at the 0.05 level). *, dominant model.

One SNP each in *PITX2*, collagen V subunit A1 (*COL5A1*), *AVGR8*, and *ZNF469* were also associated with a thinner CCT with nominal *p*<0.05. All five zinc finger E-box-binding homeobox 1 (*ZEB1*) SNPs tested were associated with modest increases in CCT, from 7 to 10 µm per minor allele. Three SNPs in solute carrier 4A11 (*SLC4A11*) had somewhat larger apparent effect sizes. The minor T allele of SNP rs6084312 was associated with an increase of about 14 µm, whereas those of rs6051669 and rs3803955 were associated with decreases of about 9 and 11 µm, respectively.

These associations generally persisted with no loss of significance when CCT was adjusted for FECD severity grade, with one important exception: evidence for association at rs613872 in *TCF4* was reduced sharply with addition of FECD grade as a covariate, and the magnitude of the estimated per-allele effect fell significantly (effect  = −0.4 µm, vs. 18.6 µm). Otherwise, the magnitude of effect sizes were overall maintained in the most strongly associated SNPs. One SNP, rs3803955 in *SLC4A11*, reached borderline study-wide significance (*p* = 0.0012; Bonferroni-corrected *p* = 0.058).

### Haplotype-based analyses

Linkage disequibrium (LD) was extensive enough among SNPs in the same gene to warrant haplotype-based association analysis (Fig. S1). We conducted both LD-based analyses of SNPs within blocks of tight LD, and moving-window analyses with a window size of three SNPs. None of the analyses of FECD case/control status, FECD severity grade or CCT revealed any association peaks substantially stronger than the corresponding single-SNP analyses.

### Analysis of rare variation in *COL8A2*

Our lack of significant association between *COL8A2* SNPs and FECD phenotypes, despite several reports linking the two [13], [14], [39], [40], led us to examine population-level genetic variation in the *COL8A2* coding region. We surveyed available polymorphism data on 1086 unrelated individuals from the 1000 Genomes Project database [54] for coding variants previously discovered in studies on FECD reporting sequence data [13], [14], [20], [39], [40], [55] (Table S4). Two mutations identified as causal, Gln455Lys [13] and Leu450Trp [14], did not appear as variant loci in any of the 1000 Genomes samples (data not shown). Another missense mutation, Arg155Gln, previously associated with early-onset FECD [13], was found in East Asian samples (CHB + CHS + JPT) with variant allele frequency 6.3%, but was present in European samples (CEU + FIN + GBR + IBS + TSI) at only 0.1% (Table S4). Only two *COL8A2* coding variants, both silent, were present at a frequency of greater than 1% in Europeans: Pro586Pro [13] (2.1%) and Gly495Gly [39] (1.2%). The latter was very common in the East Asian samples (49.8%).

## Discussion

Although several rare mutations causing familial, early-onset FECD and rare variants associated with late-onset FECD have been identified [13], [14], [27], [30], [38]–[40], [56], [57], our current knowledge of the heritable causes of late-onset FECD is severely limited. No causal variant has been identified that is common in the population and that is of relatively modest effect. To date, the locus most consistently replicated in association studies on late-onset FECD has been *TCF4* [15]–[19]. In samples of European descent, highly significant association has been reported specifically with a particular SNP, rs613872, with large per-allele ORs of between 4.0 and 5.5 [15]–[17], [19], consistent with our estimate of 6.0 (Fig. 1). Because it resides in intron 3 of *TCF4*, the causal locus at or captured by rs613872 is most likely a regulatory locus and not a coding variant. Indeed, the G allele of rs613872 was absent in a small Chinese sample of FECD cases and controls, but another SNP in intron 3, rs17089887, showed significant association with an odds ratio of 2.57 for each minor allele T [18]. These findings point to either an unknown, shared causal variant in the Chinese and European populations, or a multiplicity of common variants. Linkage to a broader region encompassing TCF4 has also been reported [16], [58], strengthening the case that the association signals reflect true linkage disequilibrium between this SNP and a causal variant, although the action of *TCF4* may be independent of the FCD2 locus at chromosome 18q21.2–21.32 [17]. The gene product of *TCF4,* E2-2, is found in the developing corneal endothelium [15], but changes in the endothelial cell density associated with FECD are not apparent in carriers of the rs613872 G allele in early adulthood [59]. The precise mechanism by which E2-2 alters the structure of the cornea is unknown, but is likely to involve regulation of genes involved in cell growth and differentiation. E2-2, after binding to β-catenin, has been shown to maintain multipotency of corneal epithelial stem cells via the *Wnt1* pathway [60], and to activate *ZEB1*, a zinc-finger transcription factor which in turn is involved in dedifferentiation of epithelial cells [61] as well as mediating collagen I deposition. We have confirmed the association and strong effect of the rs613872 variant on FECD and CCT in our multi-center sample of FECD cases and controls. However, while the association with FECD persisted when the phenotype was adjusted for independent effects on CCT, the association with CCT was lost when CCT was adjusted for FECD grade, suggesting that this SNP, or the causal variant tagged by it, affects CCT via its influence on FECD severity.

In contrast, modest association between several SNPs in *COL8A2* and CCT remained when FECD severity grade was included in the analysis as a covariate, suggesting that variation in *COL8A2* may influence CCT independently of FECD. *COL8A2* has been identified as a candidate locus for CCT in a genomewide association study (GWAS), i.e., a survey of common genetic variation [24]. Two genomewide significant SNPs in *COL8A2* from this mixed-ethnic GWAS, rs96067 and rs7550047, were also associated in our FECD case/control sample, albeit less significantly (*p*>0.001). Other studies have either failed to detect association between *COL8A2* and FECD or have not detected coding variants in *COL8A2* segregating within FECD families [20], [55].

Current evidence for the role of *COL8A2* in FECD features high-penetrance, coding-sequence changes that are rare in the general population. Such mutations have often been implicated in early-onset FECD [13], [14], [39], [40], with corroborating biological evidence from transgenic mice homozygous for the Gln455Lys mutation [62]. The 1000 Genomes data suggest that these variants are rare in populations of European descent, with allele frequencies below 1%. Notably, the best established functional variants–Gln455Lys [13] and Leu450Trp [14]–appear to be absent in all of the 1000 Genomes samples. Many implicated rare variants in *COL8A2* occur in 1000 Genomes samples at a low frequency, and these warrant additional study, but more DNA sequence data from FECD affected and unaffected individuals are necessary. While the Leu450Trp [14] and Gln455Lys [13] mutations likely do play a role in early-onset FECD, as segregation with FECD has been demonstrated, segregation has not been demonstrated for any of the variants identified in late-onset FECD, the majority of which have also been identified in unaffected individuals [13], [39]. In summary, genetic variation in *COL8A2* spans a continuum of effect sizes and allele frequencies, from common variants affecting corneal thickness in normal populations to rare exonic variants causing early-onset FECD.

Our association results from genes other than *TCF4* and *COL8A2* were quite illuminating regarding FECD pathogenesis and the underlying central corneal thickness. We observed no strong associations between FECD and SNPs in any of the other candidate genes in Table 1. Because these variants (except for *SLC4A11*) were identified in samples ascertained for early-onset FECD, our lack of strong association findings in these genes is not surprising. Weak associations with FECD were observed between SNPs in *PITX2, ZEB1, AVGR8* and *ZNF469*, and although the estimated effects were substantial (as high as 0.96 FECD grade units per allele) after adjustment for CCT, none reached study-wide significance. *PITX2*, in which mutations have been identified in Axenfeld-Rieger syndrome, appears to play an important role in development of the eye and structures derived from the pharyngeal arches [63]. We also observed a modest protective effect on FECD of the minor T allele of rs1034200 in *AVGR8*, a gene not previously implicated in FECD. Little is known about the function of *AVGR8*, which belongs to the *ZNF* family of transcription factors [33]. This result provides novel evidence for pleiotropic effects of genes involved in corneal development and molecular structure in FECD and CCT. Indeed, such relationships are expected, as described above in the case of *TCF4* and *ZEB1*.

We detected SNP associations with CCT in some, but not all, genes previously implicated. *PITX2, ZEB1, ZNF469*, and *AVGR8* likely do have an effect on FECD pathogenesis, but were observed to have a much larger estimated effect on CCT, in terms of proportion of variance explained (Tables S2 and S3), which persisted after adjustment for FECD severity grade. Hence, a larger FECD sample would be expected to yield study-wide significant results, given the observed effects in this smaller sample. Our nominal (*p*<0.05) association findings with rs1034200 in *AVGR8*, three SNPs in *ZNF469* and with rs1409832 between *COL5A1* and *RXRA*, were all genomewide significantly associated with CCT in recent GWAS [24], [33]. As with *COL8A2*, these genes were associated with an 8- to 16-µm change in corneal thickness in the present study, considerably larger than, albeit consistent with, the effects observed for genomewide significant SNPs in a recent GWAS (4–6 µm) [24]. These larger estimates may be the result of differences in sample ascertainment: our sample consists of FECD controls and FECD cases who, on average, have 55 µm thicker corneas.

We also found SNPs in *SLC4A11* and *ZEB1* to be associated with CCT. A role for *SLC4A11* has been suggested via a small number of late-onset FECD patients carrying coding-sequence mutations within samples of 89 mainly Chinese late-onset FECD patients [38], and 189 Caucasian nuclear families with late-onset FECD [27], with no apparent independent effect on CCT, although association was not established in these studies. Additionally, while nonsense mutations in *ZEB1* have been shown to cause PPCD [34], [41], missense mutations have been identified in, and presumed to be causative of, a small number of FECD cases [36], [56]. While the potential role of *ZEB1* in the pathogenesis of FECD remains unclear, it has been proposed to modify FECD pathogenesis [56]. We propose that this modification may be through its effect on CCT.

The relatively large effect sizes attributable to candidate genes associated in this study with FECD and CCT suggest that they affect corneal structure. As a characteristic feature of FECD is a thickened Descemet membrane, and as increasing CCT is associated with even early grades of FECD, baseline corneal thickness prior to the progression to severe FECD is clearly important [64]. Variants in many genes change the architecture of the cornea, including the thickness and the elastic properties. Some variants only affect thickness while others cause sufficient disorganization of the structural proteins to cause widespread guttae and, therefore, FECD.

Although our results confirm the association the rs613872 variant in *TCF4* with FECD susceptibility, predictive value of the presence of the minor allele in identifying individuals at increased risk of developing FECD is limited. The minor G allele at rs613872 is very common, with a frequency of 18% in the HapMap CEU sample (as a surrogate for a general northern European population), and 19% in our control population. Given that 27% of the cases do not carry the minor allele at rs613872, and that our control sample is, on average, the same age as our cases and shows no evidence of FECD, *TCF4* risk appears neither necessary nor sufficient to develop disease. Early reports suggest that rs613872 is not the best marker in this gene across ethnic groups, and its frequency varies across the globe [16], [18], but it may be feasible to use a composite risk score encompassing multiple markers at the *TCF4* locus to predict risk. Prospective studies following individuals with susceptibility alleles in *TCF4* are warranted to determine what percentage of individuals with risk alleles will develop FECD in their lifetime. One application of such a risk score would be early identification of individuals for monitoring and interventions by physicians, although such preventative measures have yet to be developed. Such a risk score is already in practice for age-related macular degeneration, a common retinal disease with confirmed susceptibility loci that show large effect sizes similar to *TCF4* and in aggregate yield receiver operator curves with areas under the curve greater than 80% [65], [66]. Thus, a catalog of common genetic variation affecting corneal structure may be relevant to treatment of these diseases.

## Supporting Information

Figure S1 **Linkage disequilibrium patterns for genes surveyed in association analyses for FECD and CCT.** Two plots are shown for each chromosome: D' and *r* ^2^. Only SNPs with MAF ≥1% are shown.
(PDF)

Table S1 **Results from association tests for FECD case/control status.** Minor allele frequencies for FECD cases (MAF_Cases) and controls (MAF_Controls) are included for each SNP. Only SNPs with MAF ≥1% in the combined sample are shown. Odds ratios (OR), 95% confidence intervals (95% CI) and *p* values are listed for additive and dominant inheritance models and two statistical models: FECD status adjusted for age and sex (FECD ∼ age, sex) and FECD adjusted for age, sex and average CCT (FECD ∼ age, sex, avg. CCT).
(XLS)

Table S2 **Results from association tests for FECD severity grade** (**worse eye**)**.** Statistical models are FECD severity grade adjusted for age and sex (FECD Grade ∼ age, sex) and grade adjusted for age, sex and CCT in the eye with more advanced FECD (FECD Severity Grade ∼ age, sex, worse CCT). The percentage trait variance accounted for by each SNP (perc. var.), effect size in FECD grade units (Effect), 95% CI and *p* value are given for each test.
(XLS)

Table S3 **Results from association tests for CCT, adjusted for age and sex** (**CCT ∼ age, sex**) **or for age, sex, and FECD severity grade** (**worse eye**) (**CCT ∼ age, sex, FECD severity grade**)**.** Effect size is given in µm; otherwise, quantities are as in Table S2.
(XLS)

Table S4 **Survey of the 1000 Genomes** (**October 2011**) **and Exome Variation Server** (**EVS**) **variant data on** ***COL8A2*** **coding variants previously reported in the literature.** Populations included are (list; including which make up European, African, East Asian, Hispanic). Data are grouped by variant, and at the top of each table are listed the physical map position (build 37) and the major and minor alleles (in European samples). 1000 Genomes data include numbers of individuals with 0 (m0), 1 (m1) and 2 copies of the minor allele, total major and minor alleles, and the minor allele frequency (MAF). EVS data include minor allele frequency in African Americans (AA) and Caucasians.
(XLS)
