# Supplementary material for: Differing Roles for TCF4 and COL8A2 in Central Corneal Thickness and Fuchs Endothelial Corneal Dystrophy
Source: PLoS One. 2012 Oct 23;7(10):e46742. doi: 10.1371/journal.pone.0046742 (PMC3479099; doi:10.1371/journal.pone.0046742)

Chromosome 1, *COL8A2*

D′

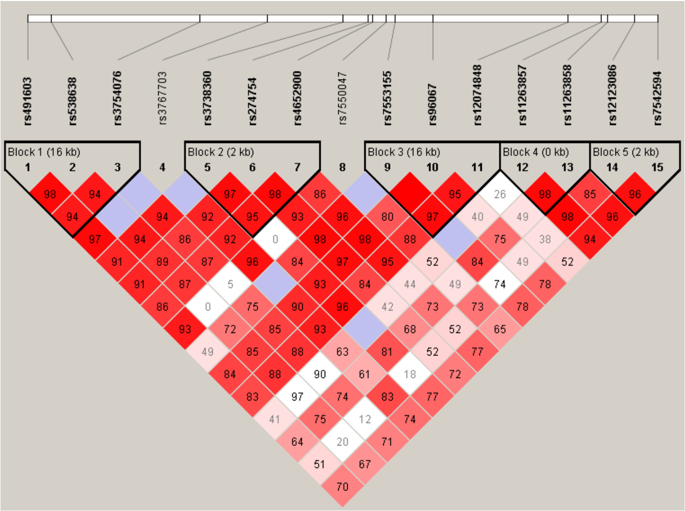

r^2

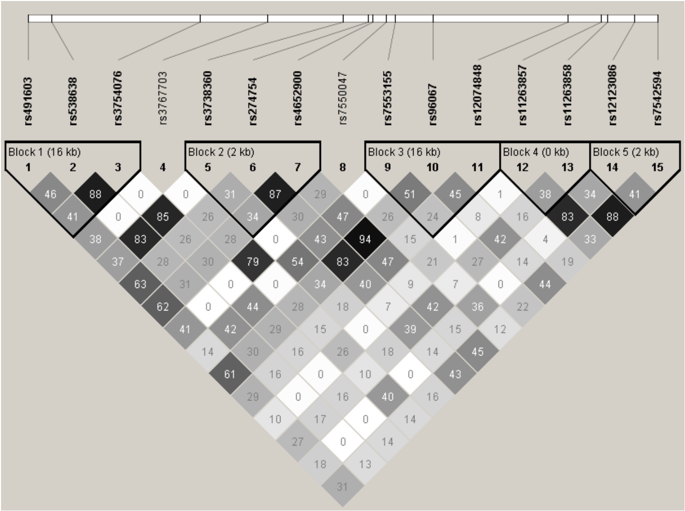

Chromosome 4, *PITX2*

D′

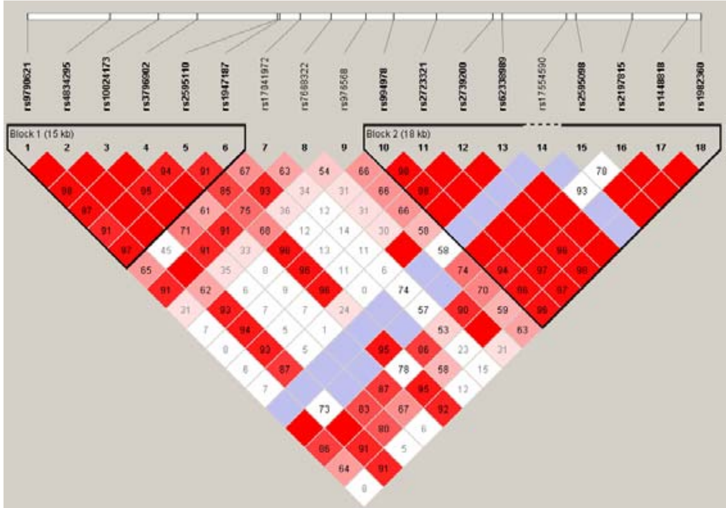

r^2

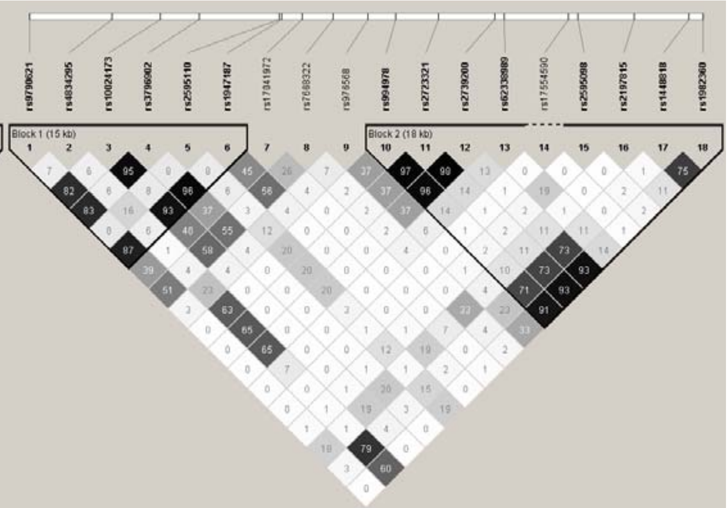

Chromosome 9, *COL5A1*

D′

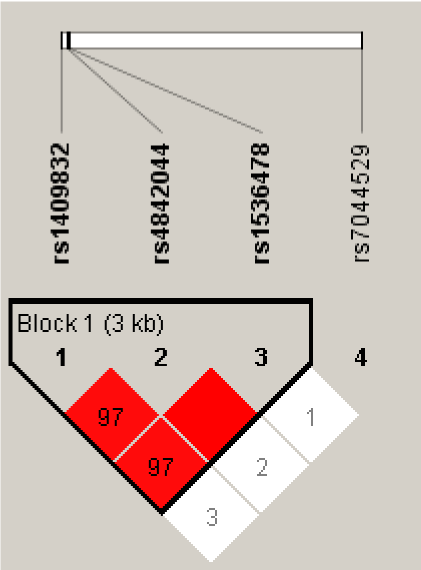

r^2

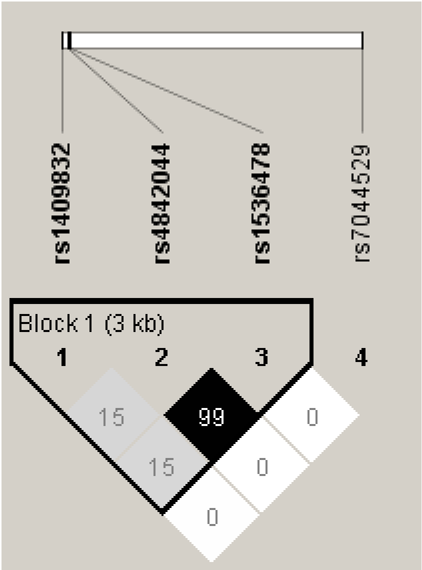

Chromosome 16, *ZNF469*

D′

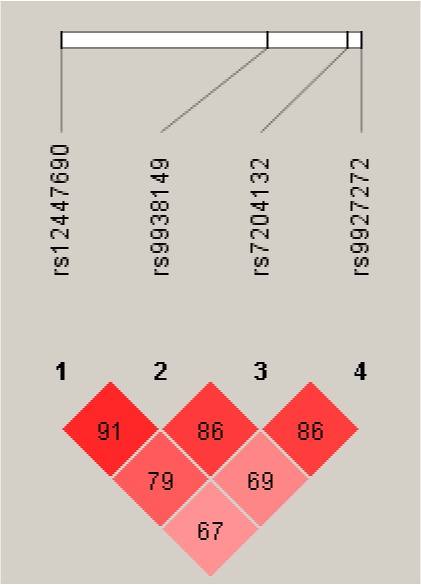

r^2

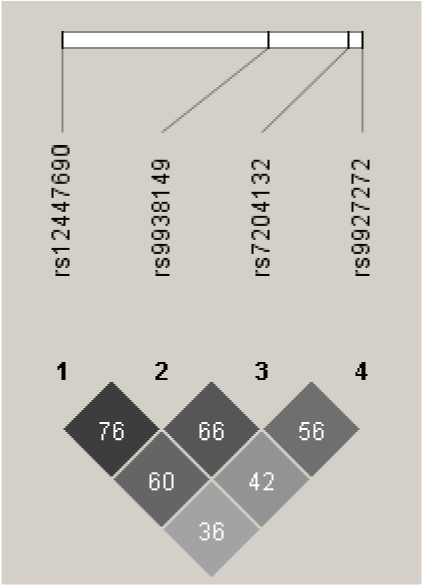

Chromosome 10, *ZEB1*

D′

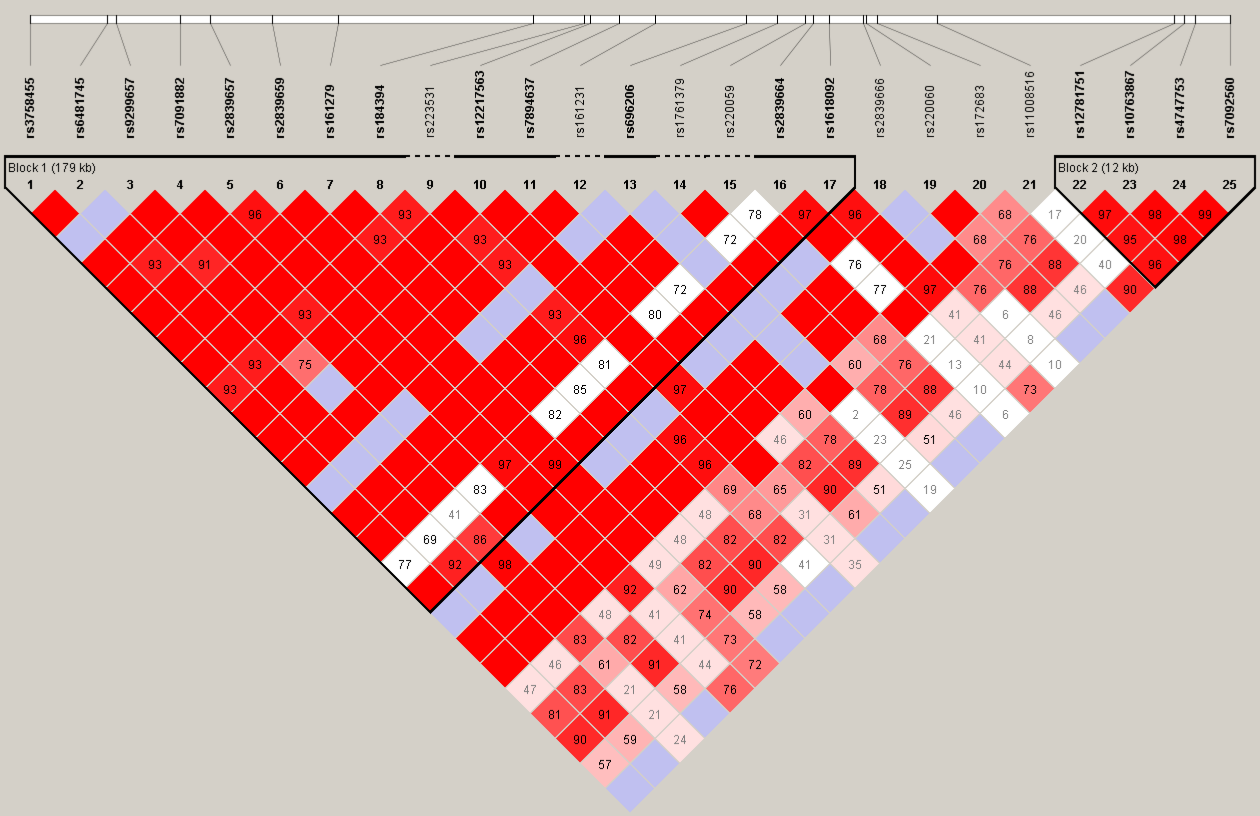

r^2

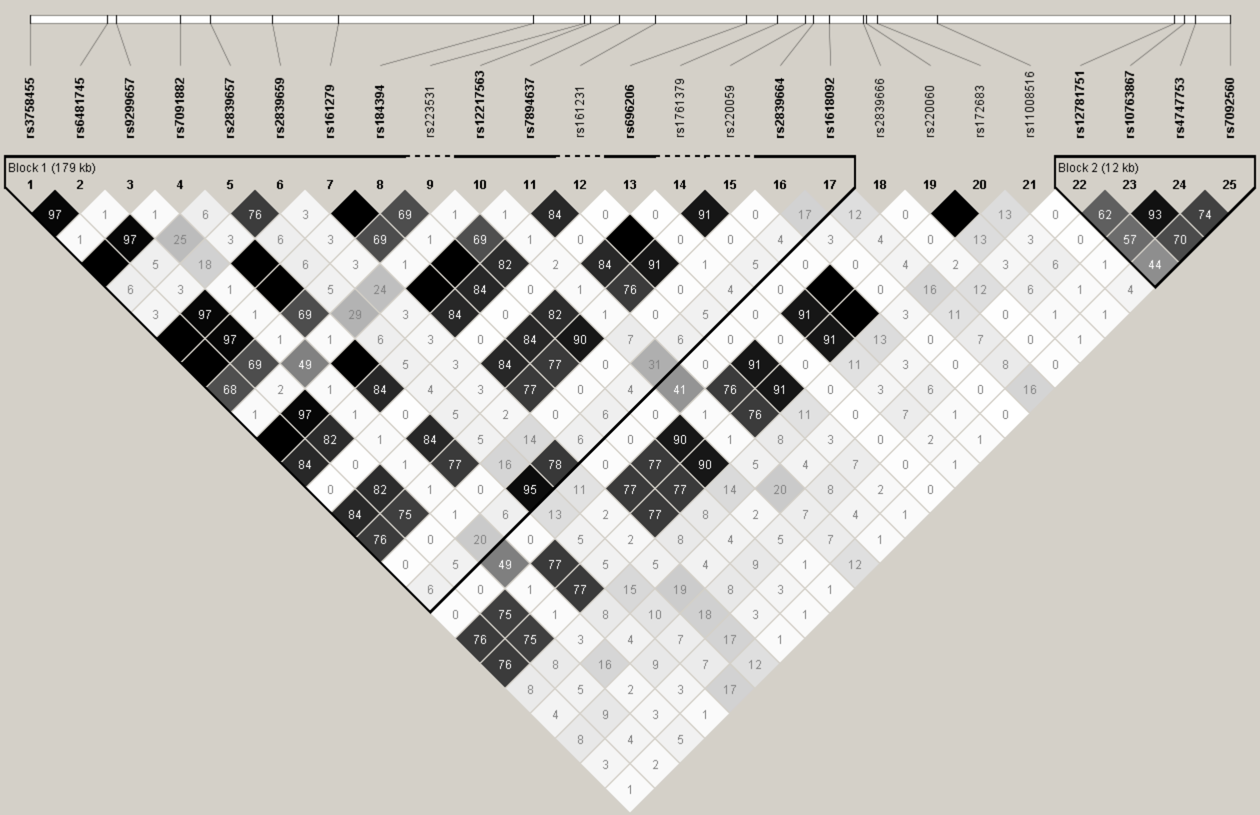

Chromosome 20, *SLC4A11*

D′

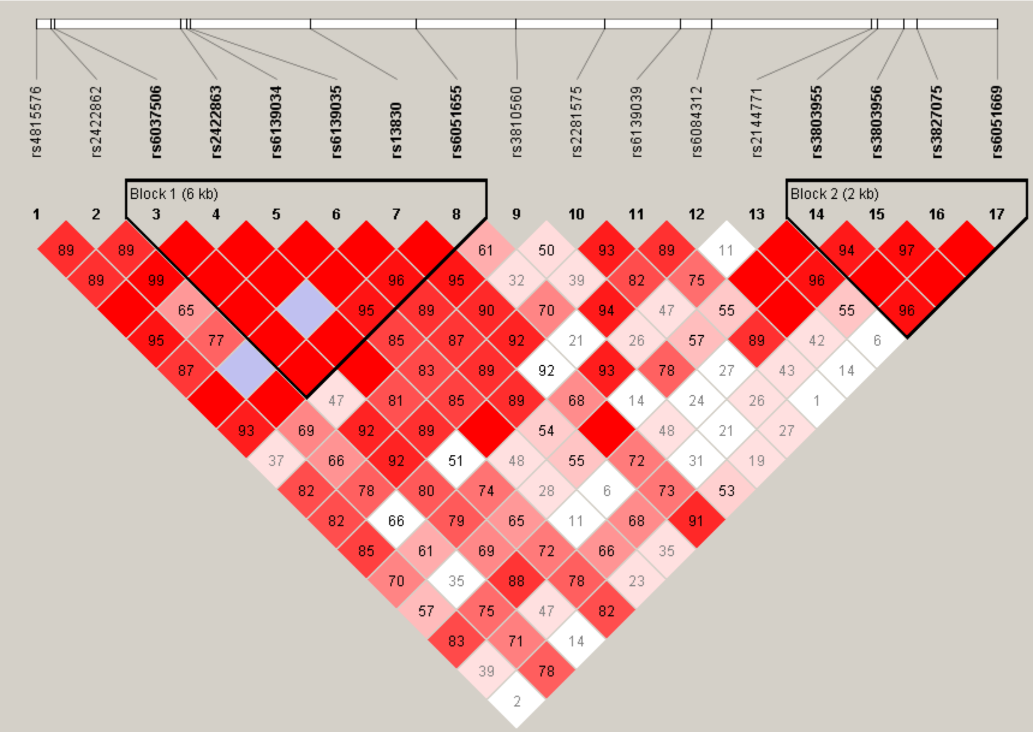

r^2

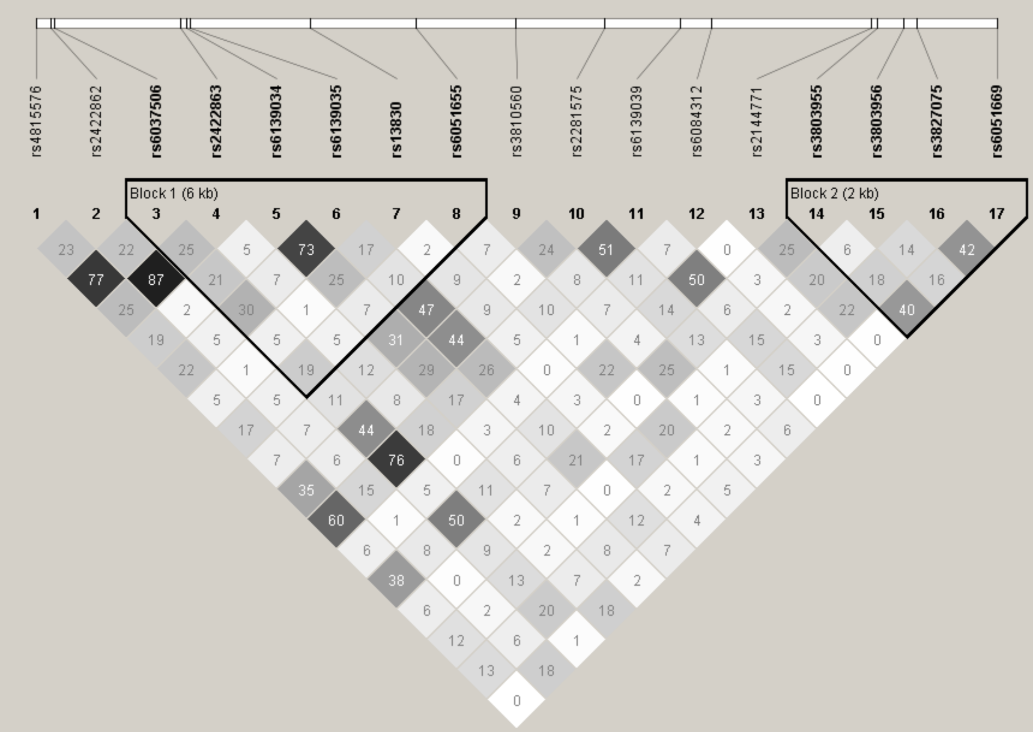

Supplement: Figure S1 — Linkage disequilibrium patterns for genes surveyed in association analyses for FECD and CCT. Two plots are shown for each chromosome: D' and r 2. Only SNPs with MAF ≥1% are shown. (PDF) [file pone.0046742.s001.pdf]
